# Neutrophil CD64 index as a new early predictive biomarker for infected pancreatic necrosis in acute pancreatitis

**DOI:** 10.1186/s12967-024-04901-9

**Published:** 2024-02-29

**Authors:** Xiangping Huang, Ling Wu, Qianhui Ouyang, Ying Huang, Lanhui Hong, Sixiang Liu, Kongzhi Yang, Ding Ning, Chao Chao Tan

**Affiliations:** 1 Department of Clinical Laboratory, The First Affiliated Hospital of Hunan Normal University (Hunan Provincial People’s Hospital), 61 Jiefang Road, Changsha, 410005 Hunan People’s Republic of China; 2 Department of Emergency, The First Affiliated Hospital of Hunan Normal University (Hunan Provincial People’s Hospital), Changsha, Hunan China; 3 Department of Emergency Medical, The Affiliated Changsha Central Hospital, Hengyang Medical School, University of South China, Changsha, Hunan China; 4 Tumor Immunity Research Center of Hunan Provincial Geriatric Institute (Hunan Provincial People’s Hospital), Changsha, Hunan China; 5 Department of Clinical Laboratory, Hunan Provincial People’s Hospital (The First Affiliated Hospital of Hunan Normal University), Changsha, Hunan China

**Keywords:** Neutrophil CD64 index, Infected pancreatic necrosis, Acute pancreatitis

## Abstract

**Objective:**

Infectious pancreatic necrosis (IPN) is a serious complication of acute pancreatitis, and early recognition and timely intervention are the keys to improving clinical outcomes. The purpose of this study was to investigate the predictive capacity of the neutrophil CD64 index (nCD64 index) on IPN in patients with acute pancreatitis

**Methods:**

This study comprises two independent cohorts: the training cohort consisted of 202 patients from Hunan Provincial People's Hospital, and the validation cohort consisted of 100 patients from Changsha Central Hospital. Peripheral blood samples were collected on the day of admission and on the 3rd, 5th, 7th, and 10th days of hospitalization, and the nCD64 index was detected by flow cytometry. Additionally, relevant clinical characteristics and laboratory biomarkers were collected and analyzed.

**Results:**

We observed that nCD64 index on admission was significantly higher in the IPN group than Non-IPN group (p < 0.001). In the training cohort, a higher occurrence rate of IPN was observed in the high nCD64 index group compared to the moderate and low nCD64 index group (p < 0.001). Further analysis showed that nCD64 index was significant positive correlated with the incidence rate of IPN (p < 0.001, correlation coefficient = 0.972). Furthermore, logistic regression analysis showed that high expression of the nCD64 index on admission was a risk factor for the occurrence of IPN (OR = 2.971, p = 0.038). We further found that the nCD64 index of IPN patients was significantly higher than the Non-IPN patients on the days 1, 3, and 5 after admission, and the nCD64 index of IPN patients before and after the onset (p < 0.05). At the same time, this study revealed that the nCD64 index on admission showed good predictive efficacy for IPN (AUC = 0.859, sensitivity = 80.8%, specificity = 87.5%), which was comparable to APACHE II score. And this finding was further validated in an independent cohort of 100 participants (AUC = 0.919, Sensitivity = 100.0%, Specificity = 76.6%).

**Conclusion:**

This study demonstrated the clinical value of nCD64 index in patients with IPN patients for the first time through two independent cohort studies. The nCD64 index can be used as an early prediction and risk assessment tool for the occurrence of IPN, contributing to the improvement of patient outcomes and efficiency of medical resource allocation.

**Supplementary Information:**

The online version contains supplementary material available at 10.1186/s12967-024-04901-9.

## Introduction

Acute pancreatitis (AP) is a common and destructive inflammatory condition of the pancreas. In recent years, with its incidence steadily increasing due to improved living standards and dietary alterations [1]. The clinical course of acute pancreatitis can be divided into two overlapping stages: early and late phases. Infected pancreatic necrosis (IPN) are the major causes of death in the late stages of patients with acute pancreatitis (up to 30%) [2–4]. Early prediction of IPN contributes to timely clinical intervention in high-risk patients, improving patient outcomes and preventing further disease progression [5].

Currently, the main approach of early diagnosing and predicting IPN involves the use of clinical scoring systems and conventional laboratory markers, such as C-reactive protein (CRP), and procalcitonin (PCT) [6–10]. Although, these clinical scores have demonstrated good performance in predicting IPN, there are still limitations in clinical application [6, 10]. Firstly, the accuracy of scoring requires the collection of a large amount of physiological and clinical data, which involves a time-consuming evaluation process. Secondly, the subjectivity scoring criteria may lead to difference in the evaluation of the score by different physicians. In addition, previous studies have shown that limited predictive efficacy of certain laboratory parameters for IPN. The area under the curve (AUC) is commonly used to quantitatively assess the overall performance of indicators [11, 12]. For example, in these studies, the AUC for CRP was only 0.597 [10]. Therefore, there are currently no effective predictors available to analyze clinical outcomes in patients with IPN. Early prediction of IPN remains a challenge.

Currently, researchers have proposed that the Neutrophil CD64 index (nCD64 index) may be a promising indicator for diagnosing infectious complications in acute pancreatitis [13]. CD64 is the monoclonal antibody which recognizes FcγR1 neutrophilic receptor, is a high affinity receptor present on neutrophils for Fc part of immunoglobulin-G (IgG) heavy chain, constitutively presents on macrophages, monocytes, and eosinophils, and only to a small extent on resting neutrophils [14]. Once infection occurs, CD64 molecules are activated by inflammatory factors, leading to a rapid increase in CD64 expressions on the surface of activated neutrophil within 4–6 h [15]. Therefore, the expression level of CD64 molecules on neutrophils can serve as an indicator of the body's infection status under inflammatory stimulation [16]. It is worth mentioning that the immune monitoring of neutrophil-related parameters is an overlooked and missing aspect of intensive care for critically ill patients [17]. The development of novel technologies and new indicators centered around neutrophils may pave the way for personalized care and a more targeted use of antibiotics in infectious diseases [17, 18]. We use keywords such as neutrophil CD64 index, infected pancreatic necrosis, acute pancreatitis, and others to search for relevant reference literature. However, there are no available studies that investigates the value of nCD64 index in predicting IPN. Previous studies have shown that the nCD64 index can be a valuable biomarker for predicting diseases such as sepsis and bacterial infections. Additionally, it can be utilized to monitor disease progression in patients with infectious inflammation [19–21]. To the best of our knowledge, only one study has investigated the diagnostic value of nCD64 index in detecting abdominal infection in acute pancreatitis [22]. However, this study is limited by its single-center design, which may result in a restricted patient population. Additionally, the study did not track the expression of nCD64 index during the progression of acute pancreatitis or further validate the research findings. Based on the aforementioned research findings, we propose the hypothesis that the nCD64 index may serve as a valuable predictive biomarker for the occurrence of IPN.

Our study employed two independent prospective cohorts to investigate the ability of nCD64 index on admission to predicting IPN and compared it with conventional biomarkers. Concurrently, we monitored the changes in nCD64 index as IPN progressed. It is evident that a blood biomarker on admission would be of great clinical importance to timely intervention and risk stratification manage of patients with IPN in acute pancreatitis. Early prediction and active medical intervention for patients with IPN contribute to reducing the risks of IPN and its complications, improving prognosis, decreasing the incidence of surgery, and enhancing the efficiency of medical resource allocation.

## Study subjects and methods

### Sample size estimation and study design

**Fig. 1:**
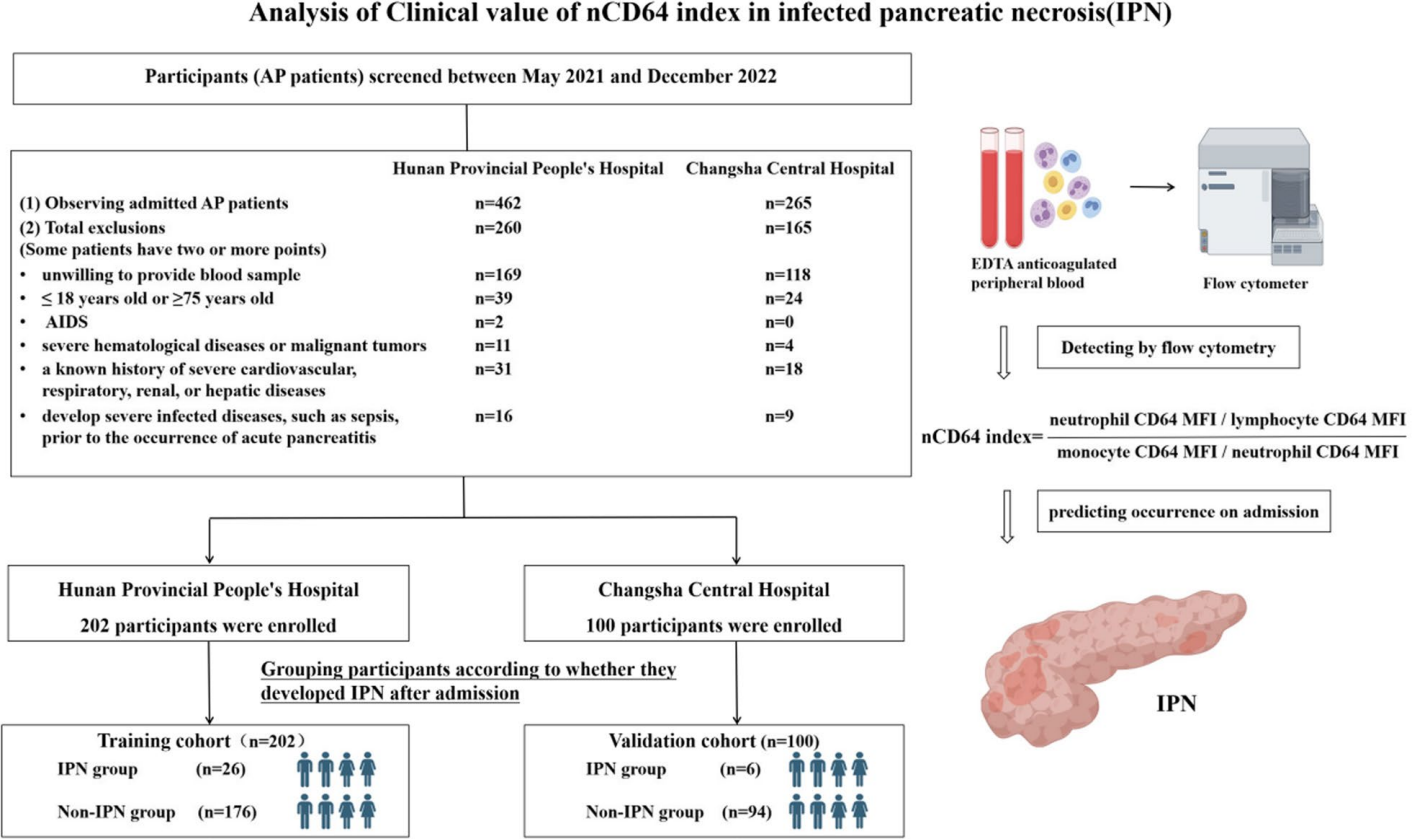
Study Flow. The recruitment period for eligible participants in this study was from May 2021 to December 2022. A total of 462 AP patients were observed at Hunan Provincial People's Hospital, among which 260 participants were excluded based on the exclusion criteria. Ultimately, 202 patients were enrolled as the training cohort. Similarly, at Changsha Central Hospital, 100 participants were identified as the validation cohort. Acute pancreatitis patients are classified into different groups based on the presence or absence of IPN occurrence after admission

This study recruited a total of 202 participants from May 2021 to December 2022 at Hunan Provincial People's Hospital as the training cohort. Additionally, 100 AP patients were enrolled from Changsha Central Hospital as the validation cohort (see Fig. 1 for details). Additional information can be found in the Additional file 1 for further reference.

For the training cohort, the sample size was estimated based on preliminary experiments and relevant literature. A one-tailed α of 0.05 and (1−β) of 0.80 were chosen. The Initial experimental group (n = 40) exhibited a sensitivity of 0.93, and the target sensitivity was set at 0.7. Based on these parameters, the calculated sample size for positive samples is 18 cases. Considering that positive cases make up for approximately 10% of all samples, a total sample size of 180 cases was required. Anticipating a follow-up loss of 10%-15%, the final determined sample size was 202 cases. Additionally, 100 participants were recruited for the validation cohort.

Within 24 h of admission, Venous blood was collected from all patients for measurement of nCD64 index by flow cytometry. Additionally, relevant clinical characteristics and laboratory biomarkers were collected and analyzed. In the training cohort, we further tracked the nCD64 index, CRP, and PCT of severe patients at the third, fifth, seventh, and tenth days after admission, as well as measured the changes in nCD64 index before and after the occurrence of IPN. This study complied with the ethical guidelines of the Declaration of Helsinki, and informed consent was obtained from all patients. The study was approved by the Medical Ethics Committee of Hunan Provincial People’s Hospital [(2022-)-21] and the Ethics Committee of Changsha Central Hospital (R201925).

### Research subjects

The diagnostic criteria for patients with acute pancreatitis (AP) included meeting at least two of the following three criteria: (1) persistent upper abdominal pain; (2) serum amylase and/or lipase concentrations of at least three-fold higher than the upper limit of normal; and (3) abdominal imaging findings consistent with AP [2, 23].

The diagnostic criteria for the IPN group included: (1) presence of pancreatic necrosis or positive tissue culture for peripancreatic necrosis; and (2) imaging evidence peripancreatic vacuoles or gas [24, 25].

The inclusion criteria were as follows: (1) meeting the diagnostic criteria for AP; (2) having complete clinical information; (3) provision of informed consent; and (4) being between the ages of 18 and 75 years.

The exclusion criteria comprised the following: (1) patients diagnosed with chronic pancreatitis or pancreatic cancer; (2) patients with pre-existing immune system disorders, including immunodeficiency diseases such as AIDS, or autoimmune disorders such as systemic lupus erythematosus, rheumatoid arthritis, and systemic vasculitis; (3) patients with severe hematological diseases or malignant tumors; (4) patients with a documented history of severe cardiovascular, respiratory, renal, or hepatic diseases; (5) patients who developed severe infectious diseases, such as sepsis, abdominal infections, or intracranial infections, prior to the onset of acute pancreatitis; (6) patients currently undergoing treatment with medications known to impact neutrophils, such as glucocorticoids, anti-thyroid medications.

The all patients with acute pancreatitis were divided into the infected pancreatic necrosis (IPN) group and non-infected pancreatic necrosis (Non-IPN) group based on whether they developed infected pancreatic necrosis after admission.

The AP patients could be classified into the mild acute pancreatitis (MAP), moderately severe acute pancreatitis (MSAP), and severe acute pancreatitis (SAP), according to the 2012 revision of the new Atlanta Classification [25, 26].

### Experimental method, main reagents and instruments

Whole blood samples were collected using EDTA anticoagulant and the nCD64 index was detected within 2 h by flow cytometry (Myriad BriCyteE6, Mindray Medical, Shenzhen, China). The reagents for nCD64 index detecting included PerCP Anti-Human CD14, PerCP Anti-Human CD45, PerCP Anti-Human CD64 (BioLegend, San Diego, CA USA). The nCD64 index detection process was outlined below: In an experimental tube, add 50 μL of whole blood sample with EDTA anticoagulant, along with 5 μL each of CD14, CD45, and CD64 antibodies. Thoroughly mix the contents of the tubes and incubate in the dark for 15 min. The next step is to lyse the red blood cells in the sample, process it, and perform flow cytometry analysis. The mean fluorescence intensity (MFI) of CD64 expression on neutrophils, lymphocytes, and monocytes in experimental tube were detected. I will provide a detailed description of the experimental procedure and gating strategy in the Additional file 1.

nCD64 index = (neutrophil CD64 MFI / lymphocyte CD64 MFI)/ (monocyte CD64 MFI / neutrophil CD64 MFI). The calculation of nCD64 index considered the intrinsic inter-individual factors to ensure measurement stability and accuracy (Due to the high and stable expression of CD64 on the surface of monocytes, it can be utilized as a positive control for nCD64 index. Conversely, CD64 is lowly expressed on the surface of lymphocytes, serving as a negative control [19, 27]).

Interleukin-2 (IL-2), Interleukin-4 (IL-4), Interleukin-6 (IL-6), Interleukin-10 (IL-10), tumor necrosis factor (TNF), interferon (IFN) were detected by Multiplex microsphere flow immunofluorescence luminescence assay (Myriad BriCyteE6, as the aforementioned). The test sample was plasma separated from EDTA-anticoagulated venous blood samples by centrifugation at 1000*g* for 10 min, and the test kit was a 6-cytokine kit (Raisecare, Shandong, China). We also collected relevant clinical characteristics and laboratory test results. PCT and CRP were detected by Mérieux VIDAS fully automated immunoassay analyzer (Bio Mérieux, Lyon, France) and Special Protein Instrument PA990 (Lifotronic Technology, Shenzhen, China), respectively. White blood cells (WBC), immature granulocyte (IG) percentage, and Neutrophil (N) count were detected by XN Blood Analysis Line (SYSMEX, Kobe, Japan) and their supporting reagents.

### Statistical analysis

The relevant clinical data, laboratory test results, and nCD64 index were analyzed using SPSS 23.0 and MedCalc software based on the grouping information in Fig. 1. The normality of continuous variables was evaluated utilizing SPSS. For measurement data conforming to a normal distribution, means ± standard deviation (x ± s) were used to analyse. Comparisons between multiple groups were conducted using one-way analysis of variance (ANOVA), while t-tests were used for comparisons between two groups. For groups with skewed distributions that could be transformed into normal distributions, transformed data were analyzed according to a normal distribution. If the data could not be transformed, the medians (quartiles) [M (QL, QU)] was utilized, and comparisons between multiple and inter-group were evaluated using Mann–Whitney U tests. Count data comparisons were conducted using chi-square (χ^2^) tests. Additionally, we conducted logistic regression analysis and Spearman's correlation analysis. Next, we utilized the medcal software to evaluate the ability of nCD64 index and other related indicators to predict the occurrence of IPN in AP patients through Receiver Operating Characteristic (ROC) curve analysis. Subsequently, we compared the ROC curves obtained from multiple indicators within the same dataset to analyze whether there were any statistically significant differences in the predictive performance of each indicator. For the joint analysis of the indicators, we first performed a binary logistic regression analysis using SPSS. Subsequently, we combined the two indicators to create a new composite index and imported it into Medcal for the aforementioned analysis. The significance threshold for all analyses was set to 0.05. Statistical significance was determined when p < 0.05.

## Results

### The comparisons of basic clinical characteristics and nCD64 index on admission between patients in IPN and Non-IPN groups

In the training cohort, there was a statistically significant difference in the nCD64 index on admission between the IPN group and the Non-IPN group (Table 1). Additionally, significant differences were also observed between the IPN and Non-IPN groups in other indicators, including SOFA (p = 0.000), APACHE II (p = 0.000), PCT (p = 0.000), and IG% (p = 0.003). Similar results were corroborated in the validation cohort, where the nCD64 index on admission was significantly higher in the IPN group (M = 2.80) compared to the Non-IPN group (M = 1.30) (refer to Additional file 1: Table S1). In addition, we compared the relevant clinical data such as gender, age, and expression of nCD64 index between the training and validation cohorts and found no differences (refer to Additional file 1: Table S2).

**Table 1:** Comparison of clinical characteristics between IPN group and Non-IPN group in the training cohort

Index	Non-IPN group (n = 176)	IPN group (n = 26)	Z/χ^2^ value	P value
Age [M (QL, QU)]	47.00 (36.00, 55.00)	40.50 (35.75, 51.25)	− 1.142	0.254
ARDS [n (%)]	23.00 (13.10)	7.00 (26.90)	3.439^*^	0.064
SIRS [n (%)]	63.00 (35.80)	20.00 (76.90)	15.830^*^	< 0.001
ICU [n (%)]	65.00 (36.90)	23.00 (88.00)	24.466^*^	< 0.001
Number of days in ICU [M (QL, QU)]	0.00 (0.00,7.00)	12.00 (5.25, 26.75)	− 5.490	< 0.001
MOF [n (%)]	24.00 (13.60)	10.00 (38.40)	9.973^*^	0.002
Male [n (%)]	133.00 (75.60)	18.00 (69.20)	0.482^*^	0.487
Local complications [n (%)]	80.00 (45.50)	21.00 (80.70)	11.301^*^	0.001
Systemic complications [n (%)]	34.00 (19.30)	15.00 (57.70)	4.873^*^	0.027
Death [n (%)]	4.00 (2.30)	4.00 (15.40)	10.239^*^	0.001
CRP [mg/L, M (QL, QU)]	51.10 (0.16, 151.51)	80.56 (27.55, 172.38)	− 1.367	0.172
WBC [ × 10^9^/L, M (QL, QU)]	10.43 (7.55, 13.82)	11.04 (8.33, 14.98)	− 0.798	0.425
N [ × 10^9^/L, M (QL, QU)]	8.24 (5.75, 11.69)	9.51 (7.18, 12.75)	− 1.333	0.182
PCT [μg/L, M (QL, QU)]	0.06 (0.05, 0.29)	0.83 (0.18, 4.01)	− 4.372	< 0.001
SOFA [M (QL, QU)]	1.00 (0.00, 3.00)	3.00 (2.00, 5.25)	− 4.529	< 0.001
APACHE II [M (QL, QU)]	4.00 (2.00,8.00)	18.50 (13.00, 24.25)	− 7.058	< 0.001
IG [%, M (QL, QU)]	0.50 (0.30, 0.70)	0.85 (0.40, 1.20)	− 2.983	0.003
nCD64 index [M (QL, QU)]	1.25 (1.01, 1.54)	2.42 (1.91, 2.86)	− 5.899	< 0.001
Etiology [n (%)]			3.381^*^	0.337
Biliogenic [n (%)]	67.00 (38.10)	8 (30.70)		
Lipogenic [n (%)]	63.00 (35.80)	9.00 (34.60)		
Alcohol [n (%)]	24.00 (13.60)	7.00 (26.90)		
Other [n (%)]	22.00 (12.50)	2.00 (7.80)		

*nCD64 index* neutrophil CD64 index, *WBC* white blood cells, *N* neutrophils, *APACHE II* acute physiological and chronic health score, *ARDS* acute respiratory distress syndrome, *SIRS* systemic inflammatory response syndrome, *MOF* multiple organ failure, *CRP* C reactive protein, *PCT* procalcitonin, *IG* immature granulocyte, *SOFA* sequential organ failure assessment, *IPN* infected pancreatic necrosis. *M (QL, QU)* median (lower quartile, upper quartile)

The “*” symbol represents results obtained through chi-square test

### The correlation between the expression of nCD64 index on admission and the occurrence of IPN

**Fig. 2:**
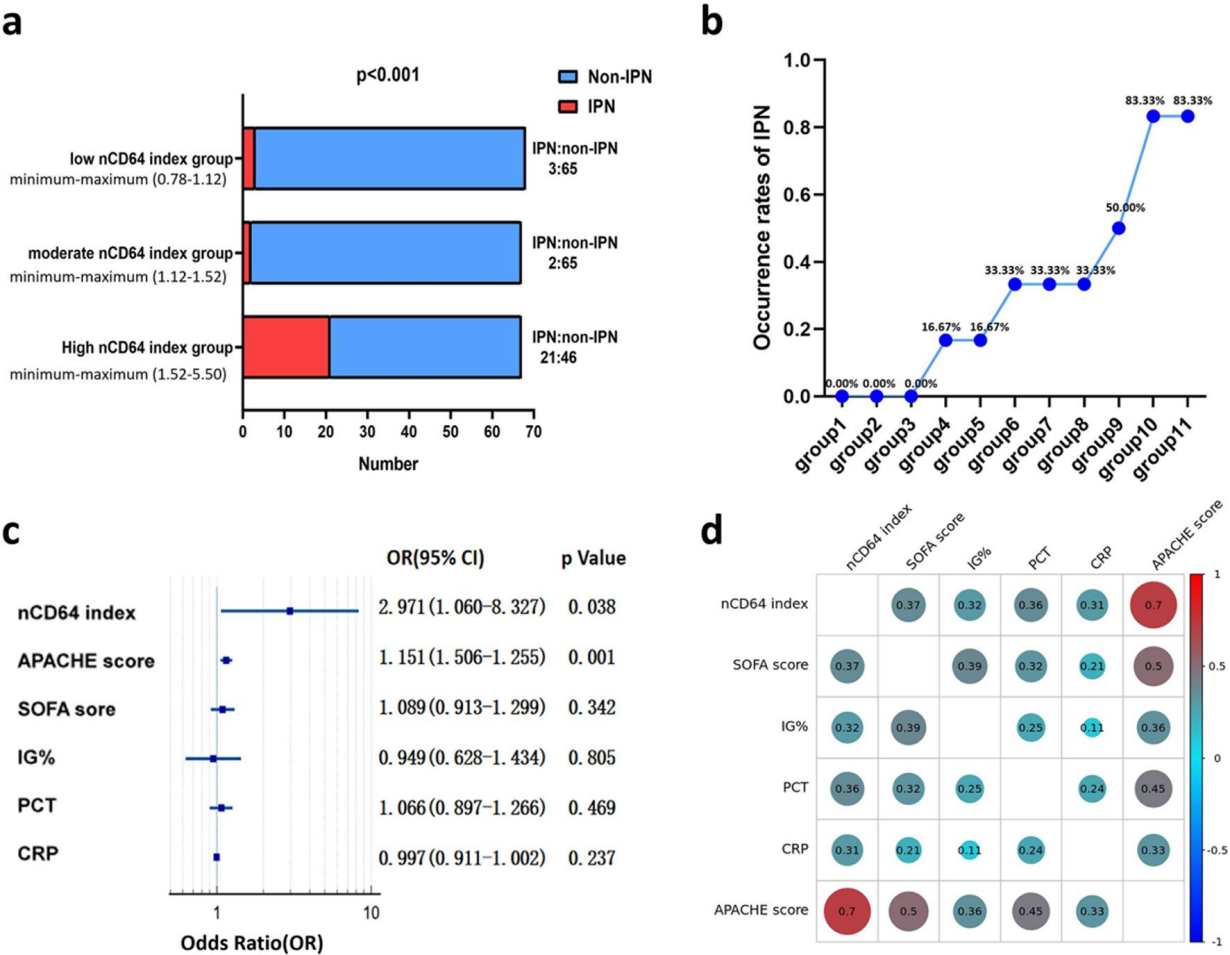
**a** The occurrence rates of IPN were observed in high, moderate and low nCD64 index group; **b** within the high nCD64 index group, based on the ranked nCD64 index from high to low, a total of 67 patients were divided into eleven group using equal frequency method to avoid data concentration in a single group; **c** to analyze the risk factors for IPN using logistic regression analysis; **d** Correlation analysis: Larger circles and darker colors indicate larger absolute values of correlation coefficients

According to the descending order of the ranked nCD64 index on admission, the AP patients were categorized into three groups using the tertile method: the high nCD64 index group (n = 67, with an IPN incidence rate of 31.34%), the moderate nCD64 index group (n = 67, with an IPN incidence rate of 2.99%), and the low nCD64 index group (n = 68, with an IPN incidence rate of 4.41%). We observed significant differences in the occurrence rates of IPN among the three groups (p < 0.001). In pairwise comparisons, there were also significant differences in the occurrence rates of IPN between the high nCD64 index group and the moderate nCD64 index group (p < 0.001), as well as between the high nCD64 index group and the low nCD64 index group (p < 0.001). However, there was no significant difference in the occurrence rates of IPN between the moderate nCD64 index group and the low nCD64 index group (p = 0.507) (Fig. 2a).

To better represent the data distribution, based on the descending order of the ranked nCD64 index on admission, we further divided the patients in the high nCD64 index group (n = 67) into eleven subgroups using the equal frequency method. This ensured that each subgroup contains an equal number of data points. For detailed information regarding the grouping, please refer to Additional file 1: Material 2. We calculated the occurrence of IPN in each group and observed an upward trend in the occurrence rates of IPN as the nCD64 index on admission increased. We further conducted a Spearman correlation analysis between the average value of each group's nCD64 index and the occurrence rates of IPN. The analysis revealed a correlation coefficient of 0.972, indicating a significantly positive correlation (p < 0.001). (Fig. 2b, Additional file 1: Table S3).

Simultaneously, logistic regression analysis demonstrated that elevated nCD64 index expression (OR = 2.971, p = 0.038) and higher APACHE score (OR = 1.151, p = 0.001) were identified as risk factors for the occurrence of IPN (Fig. 2c). Additionally, a strong correlation was observed between nCD64 index and APACHE score (p < 0.001, correlation coefficient = 0.7) (Fig. 2d, Additional file 1: Table S4).

Furthermore, this study also analyzed the correlation between the nCD64 index level on admission and the levels of inflammatory factors in patients with acute pancreatitis. The study found correlation between the nCD64 index and IL-6 (p = 0.002, correlation coefficient = 0.326), IL-10 (p < 0.001, correlation coefficient = 0.394), IL-2 (p = 0.018, correlation coefficient = 0.241) and IL-4 (p = 0.019, correlation coefficient = 0.238) (Additional file 1: Table S5).

### The predictive value of nCD64 index on admission for IPN by ROC curve analysis

We further compared the predictive value of relevant indexes in IPN patients by ROC curve analysis. The best CUT-OFF value was selected by ROC curve analysis in the training cohort, and performance was then validated using the same CUT-OFF value in the validation cohort.

The nCD64 index on admission demonstrated excellent predictive efficacy in the training cohort (AUC = 0.859, Sensitivity = 80.8%, Specificity = 87.5%) (Table 2). And the predictive efficacy of nCD64 index on admission was better than that of CRP (AUC = 0.538, Z = 3.994, p = 0.0001), IG% (AUC = 0.680, Z = 2.064, p = 0.0390), WBC (AUC = 0.549, Z = 3.550, p = 0.0004) and neutrophils (AUC = 0.581, Z = 3.269, p = 0.0011). Moreover, the predictive efficacy of nCD64 index on admission was comparable with that of APACHE II (AUC = 0.926, Z = 1.365, p = 0.1724).

**Table 2:** The predictive value of relevant indexes of IPN patients on admission in training and validation cohort

Index	AUC	Sensitivity (%)	Specificity (%)
Training cohort			
APACHE II	0.926	96.2	81.9
nCD64 index (%)	0.859	80.8	87.5
SOFA	0.769	69.2	73.3
PCT (μg/L)	0.763	76.9	68.8
IG%	0.680	50.0	84.7
CRP (mg/L)	0.583	76.9	46.6
Neutrophils	0.581	776.9	43.2
WBC	0.549	73.1	45.5
APACHE II + nCD64 index	0.934	100.0	79.6
SOFA + nCD64 index	0.904	88.5	80.1
PCT + nCD64 index	0.885	80.8	86.4
Neutrophils + nCD64 index	0.866	80.8	84.7
WBC + nCD64 index	0.860	80.8	87.5
IG% + nCD64 index	0.859	80.8	87.5
CRP + nCD64 index	0.856	80.8	89.2
Validation cohort			
APACHE II	0.924	83.3	80.6
nCD64 index (%)	0.919	100.0	76.6
SOFA	0.841	66.7	73.4
IG%	0.785	50.0	84.0
PCT (μg/L)	0.719	83.3	52.1
CRP (mg/L)	0.674	83.3	27.7
Neutrophils	0.543	66.7	61.7
WBC	0.523	50.0	75.5
APACHE II + nCD64 index	0.927	100.0	80.9
SOFA + nCD64 index	0.926	100.0	75.5
Neutrophils + nCD64 index	0.924	100.0	77.7
WBC + nCD64 index	0.922	100.0	77.7
IG% + nCD64 index	0.922	100.0	78.7
CRP + nCD64 index	0.920	100.0	76.6
PCT + nCD64 index	0.918	100.0	77.7
SOFA + nCD64 index	0.926	100.0	75.5

The collection time point for CRP, WBC, neutrophils, PCT, IG%, and nCD64 index in the Additional file 1: Table S2 was the first day of patient admission. Regarding the joint analysis of indicators, firstly, a binary logistic regression analysis was conducted to combine these two indicators, and a new composite index was created. Subsequently, a related ROC curve analysis was performed

*nCD64 index* neutrophil CD64 index, *APACHE II* acute physiological and chronic health score, *CRP* C reactive protein, *PCT* procalcitonin, *IG* immature granulocyte, *SOFA* sequential organ failure assessment, *IPN* infected pancreatic necrosis, *WBC* white blood cells

In the validation cohort, nCD64 index on admission demonstrated excellent predictive efficacy (AUC = 0.919, Sensitivity = 100.0%, Specificity = 76.6%) (Table 2). The predictive efficacy of nCD64 index on admission was better than that of CRP (AUC = 0.674, Z = 2.496, p = 0.0125), WBC (AUC = 0.523, Z = 2.2420, p = 0.0155) and neutrophils (AUC = 0.543, Z = 2.239, p = 0.0251). The predictive efficacy of nCD64 index on admission was comparable with that of APACHE II (AUC = 0.924, Z = 0.205, p = 0.8378).

Combining other indicators with the nCD64 index on admission can enhance its diagnostic capability for identifying IPN. In the training cohort the combination of CRP and the nCD64 index significantly improved predictive efficacy compared to CRP by itself (Z = 3.745, p = 0.0002). The combination of IG% and the nCD64 index significantly improved predictive efficacy compared to IG% by itself (Z = 2.113, p = 0.0346), and combining the SOFA score and nCD64 index significantly improved predictive efficacy compared to SOFA by itself (Z = 2.268, p = 0.0233). Similar results were obtained in the validation cohort, where combining traditional indexes with the nCD64 index improved predictive efficacy (Table 2).

### Multiple time point detection of nCD64 index in patients with severe pancreatitis

**Fig. 3:**
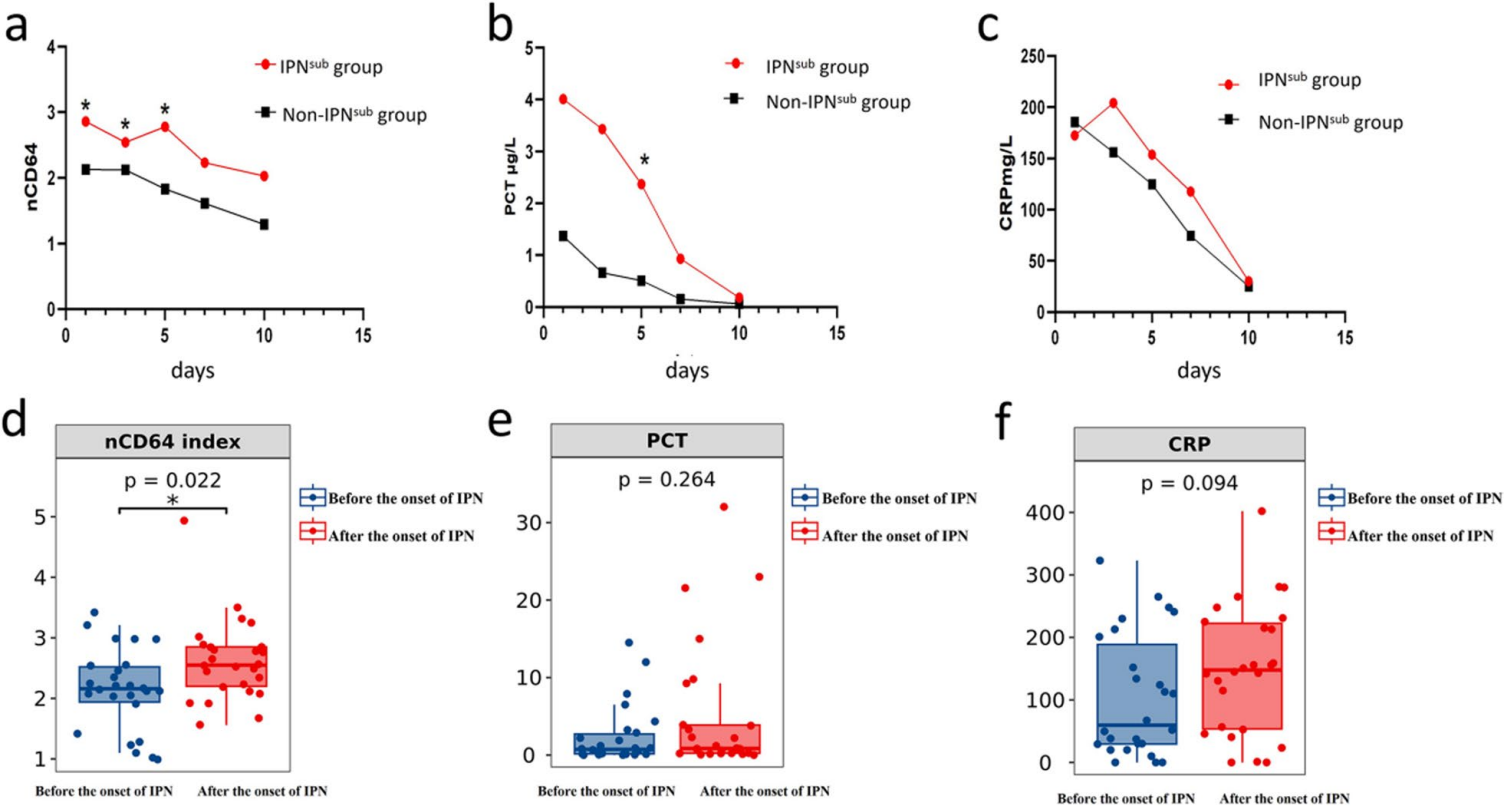
**a**–**c** The comparison of indicators between the IPN^sub^ and Non-IPN^sub^ groups in the training cohort on of the first, third, fifth, seventh, and tenth days of hospital admission. The “*” indicates that there is a significant difference between the two groups; **d**–**f** in the IPN patients of the training cohort of, a comparison of relevant indicators before and after the onset of IPN was conducted. *nCD64 index* neutrophil CD64 index, *CRP* C reactive protein, *PCT* procalcitonin, *IPN* infected pancreatic necrosis

IPN generally only occur in patients with severe pancreatitis (MSAP and SAP). During the consecutive progression of the first, third, fifth, seventh, and tenth days of hospital admission, the nCD64 index, PCT, and CRP indicators were detected in patients with severe pancreatitis. In training cohort, all patients of severe pancreatitis (n = 81) were divided into IPN^sub^ (n = 26) and Non-IPN^sub^ (n = 55) groups again based on whether they developed IPN after admission. The nCD64 index at the first, third, and fifth day after admission and the PCT at the third day 3 after admission were significantly different between patients in the IPN^sub^ and Non-IPN^sub^ groups, while the expression levels of the corresponding indexes at other time points were not significantly different (Fig. 3a–c; Additional file 1: Table S6). Statistical analysis was performed on the time points of IPN occurrence after hospital admission, revealing that the highest incidence of IPN was observed on the 5th day after admission (30.77%) (S Additional file 1: Table S7).

Additionally, we also analyzed the predictive value of nCD64 index on admission for IPN occurrence in patients with severe pancreatitis (MSAP and SAP). nCD64 index on admission also showed a good predictive efficacy that can be compared to the APACHE score (Z = 0.249, p = 0.8033) and was better than that of CRP (Z = 2.26, p = 0.0238), IG% (Z = 2.518, p = 0.0118), SOFA score (Z = 2.185, p = 0.0289). Detailed information can be found in Additional file 1: Material 8.

Furthermore, in the training cohort, the changes in relevant indicators were monitored before and after the onset of IPN in 26 patients diagnosed with IPN. The nCD64 index detected after the onset of IPN was significantly higher compared to before its occurrence (p = 0.022), while CRP and PCT showed no significant changes (Fig. 3d–f; Additional file 1: Table S9).

## Discussion

Our study is the first study aimed at exploring and validating the ability of the nCD64 index to predict the risk of IPN occurrence in patients with AP. We demonstrated that the nCD64 index on admission had excellent predictive value for IPN, comparable to the APACHE II score. Moreover, the nCD64 index also played a role in monitoring the ongoing progression of IPN. The incorporation of neutrophil CD64 index as an available blood marker upon admission proves highly significant in precisely evaluating the risk of IPN. Consequently, this assists physicians in implementing more aggressive treatment strategies and ultimately improve the clinical outcome of the patient.

Neutrophils play a pivotal role as in immune cellular barrier against pathogens, and the expression of neutrophils CD64 is a very early step of immune host response to bacterial infection [14]. However, there is currently no existing research investigating the relationship between IPN and nCD64 index. Previous studies have chiefly demonstrated the potential value of traditional biomarkers for diagnosing and predicting the occurrence of IPN, such as the APACHE II score, CRP, PCT, and other biomarkers [10, 28, 29]. In the present study, the nCD64 index on admission exhibited superior predictive efficacy for IPN compared to conventional indicators such as PCT, RCP, WBC and neutrophil count (p < 0.05). Similar findings have been reported in previous studies involving patients with sepsis and infected diseases, in which the nCD64 index demonstrated superior predictive efficacy compared to indicators such as PCT and CRP [30–32]. In recent years, the percentage of immature granulocytes (IG%) has also emerged as a new reliable clinical index for acute pancreatitis [33]. However, in the present study, the nCD64 index on admission showed better predictive efficacy compared to IG% (p < 0.05). Additionally, our study demonstrated that combining traditional markers with the nCD64 index had more excellent predictive performance compared to using traditional markers alone. These findings suggested that nCD64 index could be a valuable supplementary biomarker for predicting the occurrence of IPN and aiding in the clinical management of patients with acute pancreatitis.

Furthermore, previous studies have demonstrated the excellent predictive value of clinical scores, such as the APACHE II score, for the occurrence of IPN [6, 10]. Consistently, our study also corroborated the outstanding ability of the APACHE II score in predicting IPN, and we observed that the nCD64 index exhibited comparable predictive performance to the APACHE II score (p > 0.05). Notably, Compared to the APACHE II score, the nCD64 index is a more convenient and rapid blood biomarker for predicting the occurrence of IPN in AP patients, without the need for complex calculations and measurements. Therefore, the nCD64 index can easily be integrated into routine laboratory testing for patients with acute pancreatitis, without adding an additional burden to healthcare professionals, thus enhancing medical efficiency. Furthermore, due to the nCD64 index being unaffected by subjective factors, its results are more objective and reliable, avoiding the interference of subjective factors on the assessment of the patient’s condition. In present study, our findings highlight the potential sensitivity of the nCD64 index in monitoring the progression of infection. Here, we observed that Patients in the IPN^sub^ group exhibited significantly higher levels of the nCD64 index on admission, as well as on the third and fifth days after admission, compared to the Non-IPN^sub^ group. In contrast, traditional indicators such as CRP did not show statistically significant differences in the progression of the diseases between the two groups. Additionally, our results showed significant differences in the expression of the nCD64 index before and after the onset of IPN patients. This finding further strengthens the potential utility of the nCD64 index in complementing scoring systems, such as the APACHE II score. Additionally, the nCD64 index may provide potential advantages in the continuous monitoring of patients and facilitating early diagnosis and dynamic monitoring.

The application of nCD64 as a novel biomarker may contribute to a better understanding of the disease mechanism underlying acute pancreatitis. Inflammatory factors, such as IL-2, IL-6, and IL-10, are closely associated with acute pancreatitis [34–36]. In this study, we further analyzed the correlation between the nCD64 index and the levels of these inflammatory factors. Our findings demonstrate that nCD64 index levels were positively correlated with IL-2, IL-4, IL-6, and IL-10. The results suggest that nCD64 may play a role in the occurrence of IPN through inflammatory reactions. In future research, our aim is to further investigate the underlying mechanisms of neutrophil involvement in the occurrence of IPN, to elucidate its role in disease progression, and its interplay with other biomarkers. Moreover, investigating the mechanistic basis of the nCD64 index as a biomarker in the context of pancreatitis could open new avenues for therapeutic interventions.

The present study has several strengths. Firstly, the inclusion of patients from two independent medical centers, namely Hunan Provincial People's Hospital and Changsha Central Hospital, ensures a comprehensive and representative sample. This approach enhances the generalizability of the findings and contributes to the robustness of the study. Furthermore, the detection of nCD64 index on admission was found to be an early predictor of IPN and can improve clinical prognosis. This method offers advantageous characteristics such as convenient sampling, simple determination, low subjectivity, and faster results. Finally, our study also provides valuable clues and evidence that can serve as a foundation for future research into the role of neutrophils in the occurrence of IPN in acute pancreatitis.

Despite the strengths of the present study, there are some limitations that should be considered. The nCD64 index was not measured at multiple points in patients diagnosed with MAP. There are several reasons for this limitation. Firstly, patients with MAP typically have shorter hospitalization periods, and many MAP patients are discharged from the hospital before multiple measurements could be taken. Secondly, Patients with MAP typically do not develop IPN. Lastly, tracking MAP patients may require more funding, and focusing on severe patients could be a more efficient allocation of research resources. Nevertheless, in the present study, it was found that no patients with MAP progressed to IPN cases, so this limitation did not impact the conclusions of this study. Moreover, the small sample size of the IPN group may be another limitation of our study. However, the current sample size was determined through statistical analysis, thus leading us to believe that our research conclusions are reliable. Another limitation worth mentioning in our study is the small sample size of cases with mortality, which has restricted our ability to highlight the association between CD64 and mortality. Nonetheless, the main findings of our investigation remained unaffected. In the future, we will further explore the correlation between nCD64 index and mortality.

In summary, the early recognition and timely intervention of IPN in patients with acute pancreatitis still remain a significant challenge. In this study, the nCD64 index measured on admission demonstrated commendable predictive value for IPN, comparable to the APACHE II score. More importantly, the utilization of the nCD64 index with just one single blood sample on admission for predicting the occurrence of IPN would enhance medical efficiency. The findings of this study have significant clinical implications, particularly for the management of patients with severe pancreatitis. The nCD64 index on admission can help clinicians identify patients at high risk of IPN early in their hospital stay. This could lead to more tailored treatment strategies and potentially reduce the morbidity and mortality associated with IPN.

### Supplementary Information

**Additional file 1: Figure S1.** The gating strategy. **Table S1.** Comparison of clinical characteristics between the IPN group and the Non-IPN group in the validation cohort. **Table S2.** The differences between the training cohort and the validation cohort were compared. **Table S3.** Infected Pancreatic Necrosis (IPN) occurrence rate in High nCD64 index group. **Table S4.** Correlation of nCD64 index levels and inflammatory factors on admission in patients with acute pancreatitis. **Table S5.** Correlation of nCD64 index levels and inflammatory factors on admission in patients with acute pancreatitis. **Table S6.** Comparison between the IPN and non-IPN groups, on days 1, 3, 5, 7 and 10 in the training cohort. **Table S7.** Different Time Points of Infected Pancreatic Necrosis (IPN) Occurrence. **Table S8.** The value of relevant indexes on admission for predicting the occurrence of IPN in patients with sever pancreatitis. **Table S9.** Comparison of related indicators between pre-infection and post-infection in the training cohort. **Table S10.** Comparison of related indicators between pre-infection and post-infection in the training cohort.

## Data Availability

All data generated or analysed during this study are included in this published article [and its additional file information files]. The raw data supporting the conclusions of this article will be made available by the authors, without undue reservation.

## Abbreviations

AP: Acute pancreatitis
MAP: Mild acute pancreatitis
MSAP: Moderate severe acute pancreatitis
SAP: Severe acute pancreatitis
nCD64 index: Neutrophil CD64 index
WBC: White blood cells
N: Neutrophils
APACHE II: Acute physiological and chronic health score
IG: Immature granulocyte
SOFA: Sequential organ failure assessment
ARDS: Acute respiratory distress syndrome
SIRS: Systemic inflammatory response syndrome
MOF: Multiple organ failure
CRP: C reactive protein
PCT: Procalcitonin
AUC: Area under the curve
