# Supplementary material for: Neutrophil CD64 index as a new early predictive biomarker for infected pancreatic necrosis in acute pancreatitis
Source: J Transl Med. 2024 Feb 29;22:218. doi: 10.1186/s12967-024-04901-9 (PMC10905911; doi:10.1186/s12967-024-04901-9)
Supplement: Supplementary file 1 — Additional file 1: Figure S1. The gating strategy. Table S1. Comparison of clinical characteristics between the IPN group and the Non-IPN group in the validation cohort. Table S2. The differences between the training cohort and the validation cohort were compared. Table S3. Infected Pancreatic Necrosis (IPN) occurrence rate in High nCD64 index group. Table S4. Correlation of nCD64 index levels and inflammatory factors on admission in patients with acute pancreatitis. Table S5. Correlation of nCD64 index levels and inflammatory factors on admission in patients with acute pancreatitis. Table S6. Comparison between the IPN and non-IPN groups, on days 1, 3, 5, 7 and 10 in the training cohort. Table S7. Different Time Points of Infected Pancreatic Necrosis (IPN) Occurrence. Table S8. The value of relevant indexes on admission for predicting the occurrence of IPN in patients with sever pancreatitis. Table S9. Comparison of related indicators between pre-infection and post-infection in the training cohort. Table S10. Comparison of related indicators between pre-infection and post-infection in the training cohort. [file 12967_2024_4901_MOESM1_ESM.docx]

**Additional file**

Based on our research objectives and the inclusion criteria, a total of 202 participants were recruited from Hunan Provincial People's Hospital from May 2021 to December 2022 as the training cohort. At the same time, a validation cohort comprising 100 patients with acute pancreatitis was recruited from Changsha Central Hospital. To minimize bias from different data sources, all collected samples underwent nCD64 index testing at Hunan Provincial People's Hospital. The specific reagents and instruments used are described in the main manuscript. We utilized a independent center as the discovery cohort and further validated our findings through an other independent center. This research approach allows for the assessment of the stability and consistency of the nCD64 index in different environments, thereby facilitating a better understanding of its generalizability across diverse populations. This enhances the reliability and applicability of our study results. Additionally, we controlled for confounding factors such as age and gender to ensure the reliability of our research findings.

**Experimental procedure:**

1. Obtain peripheral blood samples using EDTA anticoagulant.

2. In an experimental tube, add 50 μl of whole blood sample with EDTA anticoagulant, along with 5 μl each of CD14, CD45, and CD64 antibodies. Thoroughly mix the contents of the tubes and incubate in the dark for 15 minutes.

3. Next, add 450 μl of lysis buffer, mix well, and incubate for 10 minutes. Wash the samples with FBS, centrifuge at 350g for 5 minutes, discard the supernatant, resuspend the cells, and load them onto the flow cytometer for detection.

**The gating strategy is as follows:**

Firstly, cell debris is eliminated through SSC and FSC gating, followed by the selection of all leukocyte populations. Then, single cells are identified and selected based on FSC-A and FSC-H gating. Furthermore, SSC and CD45 gating is employed to distinguish and select neutrophil and lymphocyte populations. Additionally, monocyte populations are accurately selected using the SSC and CD14 gates. After selecting the neutrophil, lymphocyte, and monocyte populations using the gating strategy, CD64 expression levels of each cell group are analyzed, and mean fluorescence intensity (MFI) values are calculated to obtain the nCD64 index. The nCD64 index is calculated as follows: nCD64 index = (neutrophil CD64 MFI / lymphocyte CD64 MFI) / (monocyte CD64 MFI / neutrophil CD64 MFI).

In the experimental design, we implemented a comprehensive flow cytometry control process. Firstly, we adjusted the gates by setting the voltage using blank controls. Furthermore, we established single-stain single-positive controls for CD14, CD45, and CD64 to further fine-tune the compensation. This approach ensures that the experimental data analysis is not disrupted by variations in fluorescence emission wavelengths. Additionally, during gating, we utilized isotype controls to exclude background staining caused by non-specific binding.


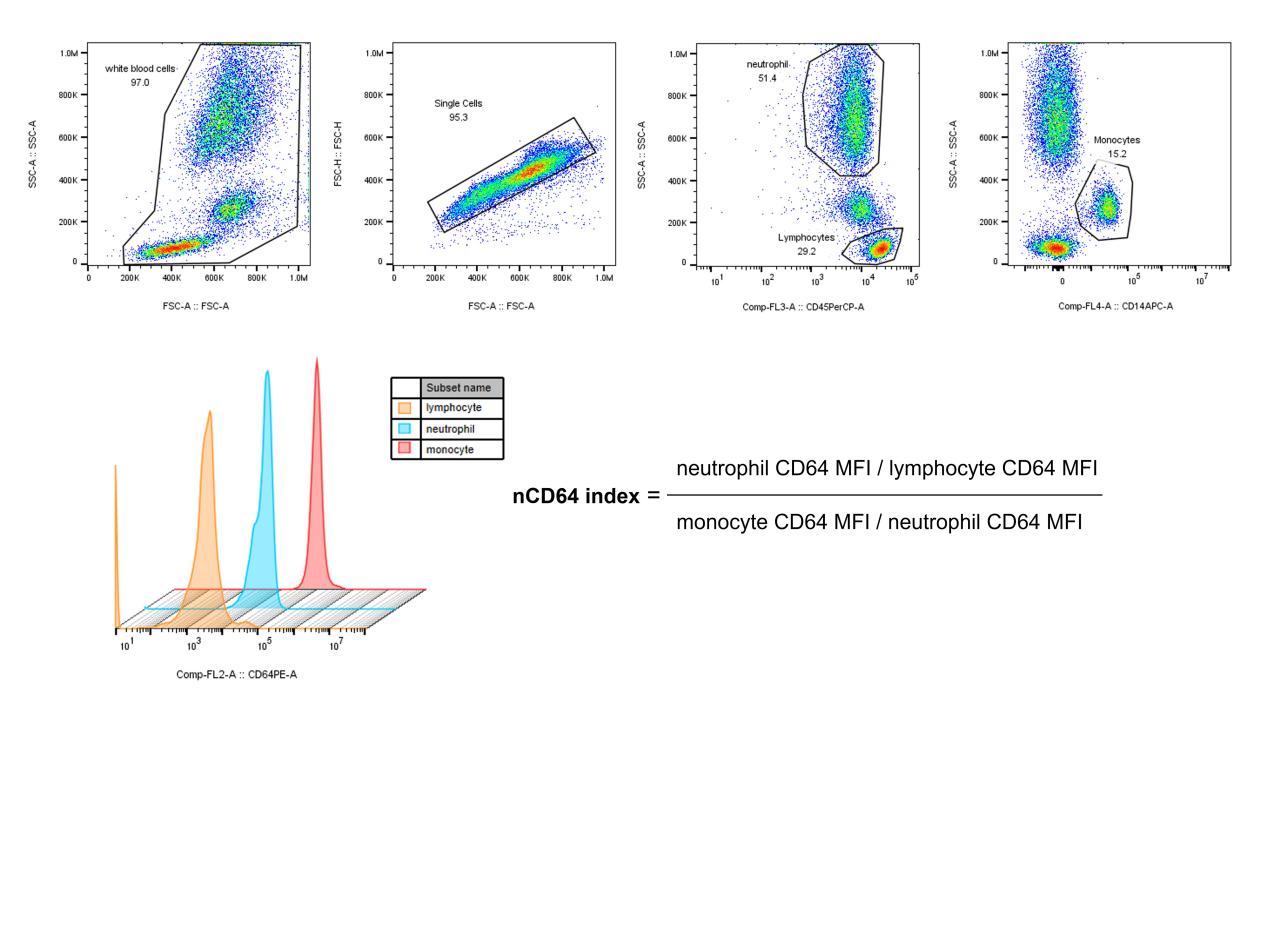


Figure S1：The gating strategy

**The rationale for nCD64 index is as follows:** In both uninfected and infected states, monocytes exhibit high expression of CD64, serving as the positive control. At the same time, lymphocytes demonstrate low expression of CD64 in both uninfected and infected states, serving as the negative control. Additionally, we have observed that non-activated neutrophils generally exhibit low expression of CD64. However, when infected, there is a significant increase in CD64 expression in neutrophils, indicating high sensitivity and specificity. The nCD64 index takes into account inherent inter-individual differences, ensuring the stability and accuracy of measurements.

(1) Additional file material 1

The collection time point for CRP, WBC, neutrophils, PCT, IG%, and nCD64 index in the Table S1 was the first day of patient admission.

Table S1: Comparison of clinical characteristics between the IPN group and the Non-IPN group in the validation cohort

| Index | Non-IPN group (n=94) | IPN group (n=6) | Z/χ2 value | P value |
| --- | --- | --- | --- | --- |
| Age〔M (QL, QU)〕 | 41.5 (34, 51) | 41.5 (33, 50) | -0.065 | 0.948 |
| APACHE Ⅱ〔M (QL, QU)〕 | 6 (3，10) | 19 (13，20) | -3.479 | 0.001 |
| ARDS〔n (%)〕 | 8 (8.5) | 2 (33.3) | 3.861 | 0.049 |
| SIRS〔n (%)〕 | 19 (20.2) | 3 (50) | 2.916 | 0.088 |
| ICU〔n (%)〕 | 20 (21.3) | 3 (50) | 2.627 | 0.105 |
| Number of days in ICU〔M (QL, QU)〕 | 0 (0，0) | 1 (0，10) | -2.253 | 0.024 |
| MOF〔n (%)〕 | 9 (9.6) | 3 (50) | 8.728 | 0.003 |
| Male〔n (%)〕 | 73 (77.7) | 5 (83.3) | 0.106 | 0.745 |
| Local complications〔n (%)〕 | 28 (29.8) | 5 (83.3) | 7.185 | 0.007 |
| Systemic complications〔n (%)〕 | 13 (13.8) | 4 (66.7) | 11.001 | 0.001 |
| death〔n (%)〕 | 0 (0.0) | 2 (33.3) | 31.972 | <0.001 |
| CRP〔mg/L, M (QL, QU)〕 | 107.44 (22.55, 175.20) | 143.58 (117.16, 259.75) | -1.423 | 0.155 |
| WBC〔×10^9^/L, M (QL, QU)〕 | 10.75 (8.24, 13.80) | 9.3 (8.13, 13.85) | -0.189 | 0.850 |
| N〔×10^9^/L, M (QL, QU)〕 | 8.69 (6.05, 11.56) | 6.81 (5.81, 11.54) | -0.348 | 0.728 |
| PCT〔μg/L, M (QL, QU)〕 | 0.18 (0.05, 0.56) | 0.85 (0.38, 10.00) | -1.796 | 0.072 |
| SOFA〔M (QL, QU)〕 | 0.5 (0, 3) | 4.5 (1.75, 7) | -2.965 | 0.003 |
| IG (%，M (QL, QU)) | 0.5 (0.3, 0.7) | 1.15 (0.6, 2) | -2.345 | 0.019 |
| nCD64 index〔M (QL, QU)〕 | 1.30 (1.03, 1.85) | 2.8 (2.08, 3.16) | -3.433 | 0.001 |
| Etiology〔n (%)〕: |  |  | 0.978 | 0.807 |
| Biliogenic〔n (%)〕 | 23 (24.5) | 2 (33.3) |  |  |
| Lipogenic〔n (%)〕 | 31 (33.0) | 1 (16.7) |  |  |
| Alcohol〔n (%)〕 | 19 (20.2) | 1 (16.7) |  |  |
| Other〔n (%)〕 | 21 (22.3) | 2 (33.3) |  |  |

Note: The nCD64 index, percentage of IG, CRP, and PCT were collected on admission. Abbreviation: Neutrophil CD64 index (nCD64 index), white blood cells (WBC), neutrophils (N), acute physiological and chronic health score (APACHE Ⅱ), acute respiratory distress syndrome (ARDS), systemic inflammatory response syndrome (SIRS), multiple organ failure (MOF), C reactive protein (CRP), procalcitonin (PCT), Immature granulocyte (IG), Sequential Organ Failure Assessment (SOFA), Infected pancreatic necrosis (IPN).

(2) Additional file material 2

There is no statistical difference between the basic information of age, gender, nCD64 index, and IPN incidence rate between the training and validation cohort.

Table S2: The differences between the training cohort and the validation cohort were compared

| index | training cohort (n=202) | validation cohort (n=100) | Z/χ2 value | P value |
| --- | --- | --- | --- | --- |
| Age〔M (QL, QU)〕 | 47（36, 54） | 41.5 (34，51) | -1.851 | 0.064 |
| Male〔n (%)〕 | 151（74.7） | 78（78） | 0.385 | 0.535 |
| nCD64 index〔M (QL, QU)〕 | 1.3（1.02, 1.72） | 1.34 (1.03，1.94) | -0.784 | 0.433 |
| WBC〔×109/L，M（QL，QU）〕 | 10.46（7.57, 13.82） | 10.63 (8.21，13.83) | -0.356 | 0.722 |
| N〔×109/L，M（QL，QU）〕 | 8.64（5.85, 11.75） | 8.46 (5.94，11.54) | -0.001 | 0.999 |
| APACHE Ⅱ〔M（QL，QU）〕 | 5 (2, 12) | 6 (3，11) | -0.264 | 0.792 |
| Etiology〔n (%)〕: |  |  | 9.400 | 0.024 |
| Biliogenic〔n (%)〕 | 75 (37.1) | 25（25） |  |  |
| Lipogenic〔n (%)〕 | 72 (35.6) | 32（32） |  |  |
| Alcohol〔n (%)〕 | 31 (15.3) | 20（20） |  |  |
| Other〔n (%)〕 | 24 (12.0) | 23（23） |  |  |
| ARDS〔n（%）〕 | 30 (14.8) | 10（10） | 1.370 | 0.242 |
| SIRS〔n（%）〕 | 83 (41.1) | 22（22） | 10.747 | 0.001 |
| ICU〔n（%）〕 | 89 (44.1) | 23（23） | 15.419 | 0.001 |
| Number of days in ICU〔M (QL, QU)〕 | 10 (0, 17.7) | 0 (0，0.75) | -3.522 | <0.001 |
| MOF〔n（%）〕 | 34 (16.8) | 12（12） | 5.129 | 0.163 |
| IPN〔n（%）〕 | 26 (12.9) | 6（6） | 3.334 | 0.068 |
| Local complications〔n (%)〕 | 102 (50.5) | 33（33） | 7.472 | 0.006 |
| Systemic complications〔n (%)〕 | 46 (22.8) | 17（17） | 0.874 | 0.350 |
| death〔n (%)〕 | 8 (3.9) | 2（2） | 0.803 | 0.370 |
| CRP〔mg/L，M（QL，QU）〕 | 229.83 (152.5, 274.73） | 109.5 (23.97，176.43) | -3.019 | 0.003 |
| PCT〔μg/L，M（QL，QU）〕 | 2.67 (0.46, 5.47) | 0.20 (0.05，0.65) | -1.462 | 0.144 |
| SOFA〔M (QL, QU)〕 | 1（0, 3） | 1 (0，3) | -0.232 | 0.823 |
| IG (%，M (QL, QU)) | 0.5（0.3, 0.8） | 0.5 (0.3，0.78) | -0.392 | 0.695 |

Note: The nCD64 index, percentage of IG, CRP, and PCT were collected on admission. Abbreviation: Neutrophil CD64 index (nCD64 index), white blood cells (WBC), neutrophils (N), acute physiological and chronic health score (APACHE Ⅱ), acute respiratory distress syndrome (ARDS), systemic inflammatory response syndrome (SIRS), multiple organ failure (MOF), C reactive protein (CRP), procalcitonin (PCT), Immature granulocyte (IG), Sequential Organ Failure Assessment (SOFA), Infected pancreatic necrosis (IPN).

(3) Additional file material 3

In the high nCD64 index group, there were a total of 67 patients. Based on the ranked nCD64 index from high to low, 67 patients were divided into eleven group using equal frequency method to avoid data concentration in a single group.

Table S3: Infected Pancreatic Necrosis (IPN) occurrence rate in High nCD64 index group

| Group (minimum-maximum) | Average value of nCD64 index on admission | Number | Number of IPN patients | Occurrence rate of IPN (%) |
| --- | --- | --- | --- | --- |
| group 1 (1.52-1.56) | 1.55 | 7 | 0 | 0.00 |
| group 2 (1.57-1.65) | 1.62 | 6 | 0 | 0.00 |
| group 3 (1.65-1.78) | 1.70 | 6 | 0 | 0.00 |
| group 4 (1.80-1.91) | 1.85 | 6 | 1 | 16.67 |
| group 5 (1.92-2.01) | 1.99 | 6 | 1 | 16.67 |
| group 6 (2.02-2.19) | 2.11 | 6 | 2 | 33.33 |
| group 7 (2.21-2.24) | 2.22 | 6 | 2 | 33.33 |
| group 8 (2.25-2.43) | 2.29 | 6 | 2 | 33.33 |
| group 9 (2.43-2.72) | 2.52 | 6 | 3 | 50.00 |
| group 10 (2.72-2.89) | 2.82 | 6 | 5 | 83.33 |
| group 11 (2.89-5.50) | 3.82 | 6 | 5 | 83.33 |
| Total | / | 67 | 21 | / |

(4) Additional file material 4

The correlation analysis of APACHE score, nCD64 index, SOFA score, IG%, PCT, and CRP in the training cohort.

Table S4: Correlation of nCD64 index levels and inflammatory factors on admission in patients with acute pancreatitis

| Correlation | | APACHE score | nCD64 index | SOFA score | IG% | PCT | CRP |
| --- | --- | --- | --- | --- | --- | --- | --- |
| APACHE sore | R | 1.000 | 0.703** | 0.505** | 0.356** | 0.448** | 0.332** |
|  | P value | / | 0.000 | 0.000 | 0.000 | 0.000 | 0.000 |
| nCD64 index | R | 0.703** | 1.000 | 0.369** | 0.317** | 0.364** | 0.314** |
|  | P value | 0.018 | / | 0.000 | 0.000 | 0.000 | 0.222 |
| SOFA score | R | 0.505** | 0.369** | 1.000 | 0.393** | 0.324** | .212** |
|  | P value | 0.000 | 0.000 | / | 0.000 | 0.000 | 0.002 |
| IG% | R | 0.356** | 0.317** | 0.393** | 1.000 | 0.254** | 0.115 |
|  | P value | 0.000 | 0.000 | 0.000 | / | 0.000 | 0.104 |
| PCT | R | 0.448** | 0.364** | 0.324** | 0.254** | 1.000 | 0.241** |
|  | P value | 0.000 | 0.000 | 0.000 | 0.000 | / | 0.001 |
| CRP | R | 0.332** | 0.314** | 0.212** | 0.115 | 0.241** | 1.000 |
|  | P value | 0.000 | 0.000 | 0.002 | 0.104 | 0.001 | / |

Note : “*” marks indicate significant correlation at the 0.05 level. “**” marks indicate significant correlations at the 0.01 level .

(5) Additional file material 5

In the training cohort, this study analyzed the correlation between nCD64 index levels and inflammatory factor levels on the first day of admission in patients with acute pancreatitis. The study found that nCD64 index levels were correlated with inflammatory factors including IL-2 IL-4, IL-6, and IL-10 (P<0.05). The correlation coefficient was 0.241 for IL-2 and 0.238 for IL-4, respectively. The correlation coefficient between nCD64 index and IL-6 was 0.326, while it was 0.394 for IL-10, confirming a moderate correlation.

Table S5: Correlation of nCD64 index levels and inflammatory factors on admission in patients with acute pancreatitis

| Correlation | | nCD64 index | IL-2 | IL-4 | IL-6 | IL-10 | TNF | IFN |
| --- | --- | --- | --- | --- | --- | --- | --- | --- |
| nCD64 index | R | 1.000 | 0.241* | 0.238* | 0.326** | 0.394** | 0.134 | 0.153 |
|  | P value | / | 0.018 | 0.019 | 0.002 | 0.000 | 0.124 | 0.093 |
| IL-2 | R | 0.241* | 1.000 | 0.650** | 0.421** | 0.781** | 0.089 | 0.590** |
|  | P value | 0.018 | / | 0.000 | 0.000 | 0.000 | 0.222 | 0.000 |
| IL-4 | R | 0.238* | 0.650** | 1.000 | 0.319** | 0.518** | 0.609** | 0.585** |
|  | P value | 0.019 | 0.000 | / | 0.002 | 0.000 | 0.000 | 0.000 |
| IL-6 | R | 0.326** | 0.421** | 0.319** | 1.000 | 0.576** | 0.006 | 0.345** |
|  | P value | 0.002 | 0.000 | 0.002 | / | 0.000 | 0.478 | 0.001 |
| IL-10 | R | 0.394** | 0.781** | 0.518** | 0.576** | 1.000 | 0.074 | 0.494** |
|  | P value | 0.000 | 0.000 | 0.000 | 0.000 | / | 0.261 | 0.000 |
| TNF | R | 0.134 | 0.089 | 0.609** | 0.006 | 0.074 | 1.000 | 0.530** |
|  | P value | 0.124 | 0.222 | 0.000 | 0.478 | 0.261 | / | 0.000 |
| IFN | R | 0.153 | 0.590** | 0.585** | 0.345** | 0.494** | 0.530** | 1.000 |
|  | P value | 0.093 | 0.000 | 0.000 | 0.001 | 0.000 | 0.000 | / |

Note : “*” marks indicate significant correlation at the 0.05 level. “**” marks indicate significant correlations at the 0.01 level.

（6）Additional file material 6

IPN generally only occur in patients with severe pancreatitis (MSAP and SAP). During the consecutive progression of days 1, 3, 5, 7, and 10 of hospital admission, the nCD64 index, PCT, and CRP indicators were detected in patients with severe pancreatitis. In training cohort, all severe pancreatitis (n=81) were divided into IPN^sub^ (n=26) and Non-IPN^sub^ (n=55) groups again based on whether they developed IPN after admission again.

nCD64-1 is the nCD64 index at day 1 after admission, nCD64-3 is the nCD64 index at day 3 after admission, nCD64-5 is the nCD64 index at day 5 after admission, nCD64-7 is the nCD64 index at day 7 after admission, and nCD64-10 is the nCD64 index at day 10 after admission.PCT, CRP are named according to the same rules.

Table S6：Comparison between the IPN and non-IPN groups, on days 1, 3, 5, 7 and 10 in the training cohort

|  | Non-IPN^sub^ group (n=55) | IPN^sub^ group (n=26) | Z value | P value |
| --- | --- | --- | --- | --- |
| nCD64 index-1〔M (QL, QU)〕 | 2.13 (1.74, 2.36) | 2.86 (2.42, 3.24) | -3.582 | 0.000 |
| nCD64 index-3〔M (QL, QU)〕 | 2.12 (1.52, 2.52) | 2.54 (2.16, 3.37) | -2.92 | 0.004 |
| nCD64 index-5〔M (QL, QU)〕 | 1.83 (1.39, 2.26) | 2.78 (2.06, 3.04) | -3.189 | 0.001 |
| nCD64 index-7〔M (QL, QU)〕 | 1.61 (1.02, 2.13) | 2.23 (1.74, 3.09) | -1.53 | 0.126 |
| nCD64 index-10〔M (QL, QU)〕 | 1.29 (0, 1.88) | 2.03 (0, 2.53) | -1.492 | 0.136 |
| PCT-1〔μg/L, M (QL, QU)〕 | 1.37 (0.31, 5.06) | 4.01 (0.83, 9.13) | -1.751 | 0.080 |
| PCT-3〔μg/L, M (QL, QU)〕 | 0.66 (0.21, 1.94) | 3.43 (0.59, 22.89) | -1.742 | 0.082 |
| PCT-5〔μg/L, M (QL, QU)〕 | 0.51 (0.09, 1.13) | 2.37 (0.53, 8.32) | -2.186 | 0.029 |
| PCT-7〔μg/L, M (QL, QU)〕 | 0.15 (0, 0.4) | 0.93 (0.02, 9.75) | -1.189 | 0.235 |
| PCT-10〔μg/L, M (QL, QU)〕 | 0.06 (0, 0.7) | 0.18 (0, 3.63) | -0.649 | 0.516 |
| CRP-1〔mg/L, M (QL, QU)〕 | 185.5 (140.1, 305.6) | 172.4 (80.6, 253.1) | -1.103 | 0.270 |
| CRP-3〔mg/L, M (QL, QU)〕 | 156 (101, 258.3) | 204 (128.5, 280.3) | -0.612 | 0.540 |
| CRP-5〔mg/L, M (QL, QU)〕 | 124.7 (56.2, 158.8) | 153.6 (24.8, 223) | -0.115 | 0.909 |
| CRP-7〔mg/L, M (QL, QU)〕 | 74.4 (1.57, 146.1) | 117.5 (0, 206.7) | -0.136 | 0.892 |
| CRP-10〔mg/L, M (QL, QU)〕 | 25.1 (0, 66.8) | 30.1 (0, 130.3) | -0.119 | 0.905 |

Note: neutrophil CD64 index (nCD64 index), C reactive protein (CRP), procalcitonin (PCT), Infected pancreatic necrosis (IPN).

(7) Additional file material 7

Table S7 presents the incidence of IPN at different time points.

Table S7: Different Time Points of Infected Pancreatic Necrosis (IPN) Occurrence

| Time Points of IPN Occurrence | Number of Cases | Daily incidence rate of IPN (%) | Cumulative incidence rate of IPN (%) |
| --- | --- | --- | --- |
| Day 1 of Hospitalization | 0 | 0.00 | 0.00 |
| Day 2 of Hospitalization | 1 | 3.85 | 3.85 |
| Day 3 of Hospitalization | 2 | 7.69 | 11.54 |
| Day 4 of Hospitalization | 6 | 23.08 | 34.62 |
| Day 5 of Hospitalization | 8 | 30.77 | 65.39 |
| Day 6 of Hospitalization | 4 | 15.38 | 80.77 |
| Day 7 of Hospitalization | 1 | 3.85 | 84.62 |
| Day 8 of Hospitalization | 2 | 7.69 | 92.31 |
| Day 9 of Hospitalization | 0 | 0.00 | 92.31 |
| Day 10 of Hospitalization | 1 | 3.85 | 96.16 |
| Day 11 of Hospitalization | 0 | 0.00 | 96.16 |
| Day 12 of Hospitalization | 0 | 0.00 | 96.16 |
| Day 13 of Hospitalization | 0 | 0.00 | 96.16 |
| Day 14 of Hospitalization | 0 | 0.00 | 96.16 |
| Day 15 of Hospitalization | 1 | 3.85 | 100.00 |
| Subsequent Time Points | 0 | 0.00 | 100.00 |
| Total | 26 | 100.00 | 100.00 |

(8) Additional file material 8

The included AP patients were classified into the MAP, MSAP and SAP groups, respectively, according to the 2012 revision of the new Atlanta Classification.Mild acute pancreatitis lacks both organ failure and local or systemic complications. Moderately severe acute pancreatitis has transient organ failure (organ failure of <2 days), local complications, and/or exacerbation of coexistent disease. Severe acute pancreatitis is defined by the presence of persistent organ failure (organ failure persists for ≥2 days).

We also analyzed the predictive value of nCD64 index on admission for IPN occurrence in severe pancreatitis (MSAP and SAP), and nCD64 index on admission also showed a good predictive efficacy that can be compared to the APACHE score. The predictive efficacy of nCD64 index on admission was comparable with that of APACHE II in training cohort (Z=0.213, P=0.8311) and validation cohort (Z=0.0937, P=0.9254). And the predictive efficacy of nCD64 index on admission was better than that of CRP in training cohort (Z=2.260, P=0.0238) and validation cohort (Z=2.161, P=0.0307). The predictive efficacy of nCD64 index on admission was better than that of IG% in training cohort (Z=2.518, P=0.0118). The predictive efficacy of nCD64 index on admission was better than that of SOFA in training cohort (Z=2.185, P=0.0289).

Table S8: The value of relevant indexes on admission for predicting the occurrence of IPN in patients with sever pancreatitis

| Index | AUC | Sensitivity (%) | specificity (%) |
| --- | --- | --- | --- |
| **Training cohort：** Non-IPN^sub^ group (n=55)； IPN^sub^ group (n=26) | | | |
| APACHEⅡ | 0.764 | 0.962 | 0.418 |
| nCD64 index (%) | 0.748 | 0.692 | 0.782 |
| PCT (μg/L) | 0.621 | 0.615 | 0.636 |
| CRP (mg/L) | 0.576 | 73.1 | 50.9 |
| SOFA | 0.529 | 69.23 | 40.0 |
| IG% | 0.524 | 50.0 | 67.27 |
| **Validation cohort :** Non-IPN^sub^ group (n=30)； IPN^sub^ group (n=6) | | | |
| APACHEⅡ | 0.821 | 0.941 | 0.4 |
| nCD64 index (%) | 0.829 | 0.706 | 0.8 |
| PCT (μg/L) | 0.738 | 83.33 | 66.67 |
| CRP (mg/L) | 0.635 | 52.4 | 83.8 |
| SOFA | 0.594 | 66.67 | 63.33 |
| IG% | 0.672 | 50.0 | 86.67 |

(9) Additional file material 9

Table S9 provides a comparison of relevant indicators before and after infection in the training cohort of IPN patients.

Table S9：Comparison of related indicators between pre-infection and post-infection in the training cohort

|  | pre-infection | post-infection | Z value | P value |
| --- | --- | --- | --- | --- |
| nCD64 index〔M (QL, QU)〕 | 2.05 (1.28, 2.35) | 2.52 (2.08, 2.76) | -2.288 | 0.022 |
| CRP〔mg/L, M (QL, QU)〕 | 110.05 (30.05, 247.88) | 143 (40.53, 213) | -1.675 | 0.094 |
| PCT〔μg/L, M (QL, QU)〕 | 0.36 (0.12, 0.92) | 0.71 (0.21, 2.2) | -1.117 | 0.264 |

Note: Abbreviation: Neutrophil CD64 (nCD64 index), C reactive protein (CRP), procalcitonin (PCT)

**Expression of nCD64 index in healthy individuals and AP patients**

In training cohort, we also included 30 healthy individuals to a healthy control group in our study. We compared the AP group (all patients with acute pancreatitis) with the healthy control group and found significant differences in the nCD64 index (Table S10).

The healthy control group's inclusion criteria were as follows: (1) no acute or chronic infectious diseases; (2) no malignant tumors or autoimmune diseases; (3) no damage to organs such as the heart, liver, or kidney damage; (4) no taking anti-inflammatory analgesic drugs; (5) no history of pancreatic-related diseases, and no elevation in serum amylase and lipase concentrations.

Table S10：Comparison of clinical characteristics between healthy controls and patients with acute pancreatitis in training cohort

| Index | Healthy control group (n=30) | Acute pancreatitis group (n=202) | Z/χ2 value | P value |
| --- | --- | --- | --- | --- |
| Age〔year, M (QL, QU)〕 | 39 (31, 52) | 47 (36, 54) | 2246.5 | 0.022 |
| Male〔n (%)〕 | 16 (53.3) | 151 (74.7) | 5.942 | 0.015 |
| nCD64 index〔M (QL, QU)〕 | 0.92 (0.86, 1.02) | 1.3 (1.02, 1.72) | 791.5 | 0.000 |
| WBC〔×10^9^/L, M (QL, QU)〕 | 6.33 (5.12, 7.10) | 10.46 (7.57, 13.82) | 868.5 | 0.000 |
| N〔×10^9^/L, M (QL, QU)〕 | 3.81 (2.82, 4.16) | 8.64 (5.85, 11.75) | 567.0 | 0.000 |
| APACHE Ⅱ〔M (QL, QU)〕 | - | 5 (2, 12) | - | - |
| Pathogenesis〔n (%)〕: |  |  |  |  |
| Biliary origin〔n (%)〕 | - | 75 (37.1) | - | - |
| Lipogenic〔n (%)〕 | - | 72 (35.6) | - | - |
| Alcoholic〔n (%)〕 | - | 31 (15.3) | - | - |
| Other〔n (%)〕 | - | 24 (12.0) | - | - |
| ARDS〔n (%)〕 | - | 30 (14.8) | - | - |
| SIRS〔n (%)〕 | - | 83 (41.1) | - | - |
| ICU〔n (%)〕 | - | 89 (44.1) | - | - |
| Number of days in ICU〔M (QL, QU)〕 | - | 10 (0, 17.7) | - | - |
| MOF〔n (%)〕 | - | 34 (16.8) | - | - |
| IPN〔n (%)〕 | - | 26 (12.9) | - | - |
| Local complications〔n (%)〕 | - | 102 (50.5) | - | - |
| Systemic complications〔n (%)〕 | - | 46 (22.8) | - | - |
| death〔n (%)〕 | - | 8 (3.9) | - | - |
| CRP〔mg/L, M (QL, QU)〕 | - | 229.83 (152.5, 274.73) | - | - |
| PCT〔μg/L, M (QL, QU)〕 | - | 2.67 (0.46, 5.47) | - | - |
| SOFA〔M (QL, QU)〕 | - | 1 (0, 3) | - | - |
| IG (%，M (QL, QU)) | - | 0.5 (0.3, 0.8) | - | - |

Note: The nCD64 index, APACHE II score, SOFA score, percentage of IG, CRP, neutrophil and PCT were collected on admission. Abbreviation: Neutrophil CD64 index (nCD64 index), white blood cells (WBC), neutrophils (N), acute physiological and chronic health score (APACHE Ⅱ), acute respiratory distress syndrome (ARDS), systemic inflammatory response syndrome (SIRS), multiple organ failure (MOF), C reactive protein (CRP), procalcitonin (PCT), Immature granulocyte (IG), Sequential Organ Failure Assessment (SOFA), Infected pancreatic necrosis (IPN).
